# The Membrane Protein LasM Promotes the Culturability of *Legionella pneumophila* in Water

**DOI:** 10.3389/fcimb.2016.00113

**Published:** 2016-09-28

**Authors:** Laam Li, Sébastien P. Faucher

**Affiliations:** Department of Natural Resource Sciences, Faculty of Agricultural and Environmental Sciences, McGill UniversityMontreal, QC, Canada

**Keywords:** *Legionella pneumophila*, freshwater, survival, *lpg1659*, membrane protein, metal transporter

## Abstract

The water-borne pathogen *Legionella pneumophila (Lp)* strongly expresses the *lpg1659* gene in water. This gene encodes a hypothetical protein predicted to be a membrane protein using *in silico* analysis. While no conserved domains were identified in Lpg1659, similar proteins are found in many *Legionella* species and other aquatic bacteria. RT-qPCR showed that *lpg1659* is positively regulated by the alternative sigma factor RpoS, which is essential for *Lp* to survive in water. These observations suggest an important role of this novel protein in the survival of *Lp* in water. Deletion of *lpg1659* did not affect cell morphology, membrane integrity or tolerance to high temperature. Moreover, *lpg1659* was dispensable for growth of *Lp* in rich medium, and during infection of the amoeba *Acanthamoeba castellanii* and of THP-1 human macrophages. However, deletion of *lpg1659* resulted in an early loss of culturability in water, while over-expression of this gene promoted the culturability of *Lp*. Therefore, these results suggest that *lpg1659* is required for *Lp* to maintain culturability, and possibly long-term survival, in water. Since the loss of culturability observed in the absence of Lpg1659 was complemented by the addition of trace metals into water, this membrane protein is likely a transporter for acquiring essential trace metal for maintaining culturability in water and potentially in other metal-deprived conditions. Given its role in the survival of *Lp* in water, Lpg1659 was named LasM for *Legionella* aquatic survival membrane protein.

## Introduction

*Legionella pneumophila* (*Lp*) is a water-borne opportunistic pathogen that can infect human alveolar macrophages, resulting in a severe form of pneumonia called Legionnaires' disease (Fields et al., 2002). Recent years have seen an increase in the incidence of sporadic cases and outbreaks of Legionnaires' disease around the world, mostly associated with *Legionella*-contaminated man-made water systems (European Centre for Disease Prevention and Control, 2013; Phin et al., 2014). Since the major mode of transmission is through water, the dynamics of *Lp* in freshwater have been studied extensively. It is known that *Lp* replicates only in the presence of sufficient nutrients or permissive hosts (Fields et al., 2002). Under nutrient limitation, *Lp* can survive for a prolonged period of time, up to 1.5 years in some cases, in varying compositions of freshwater including tap water, drinking water and creek water (Skaliy and McEachern, 1979; Schofield, 1985; Lee and West, 1991; Paszko-Kolva et al., 1992; Söderberg et al., 2008).

In an artificial freshwater model, Fraquil, *Lp* had less than a 1-log reduction in CFU counts after 20 weeks at 25°C (Li et al., 2015; Mendis et al., 2015). Moreover, *Lp* can survive for several months under a range of temperatures, pH and trace metal concentrations and remain infectious more than 6 months after exposure to Fraquil (Mendis et al., 2015). This ability for long-term survival in different kinds of water and under different conditions allows *Lp* to colonize water systems, and persist until it encounters a suitable host.

A limited number of studies have identified genes that are important for the survival of *Lp* in water. Söderberg et al. (2008) found that *lspD, lspE, lspF*, and *pilD*, which encode components of the virulence-related type II secretion system, are necessary for *Lp* to survive in water at temperatures below 17°C. In addition, the alternative sigma factor RpoS, as well as the stringent response regulators RelA and SpoT are required for the survival of *Lp* in water at 25°C (Trigui et al., 2014). In order to identify additional genes contributing to survival in water, we previously conducted a microarray analysis comparing the transcriptome of *Lp* exposed to water to that of *Lp* grown in rich medium (Li et al., 2015). Since bacteria tend to respond to environmental changes via transcriptomic reorganization (Ishihama, 2000; Hecker et al., 2009), genes that are highly up-regulated upon water exposure could be crucial for the successful adaptation and survival of *Lp* in water systems. Using this approach, we found that one highly up-regulated gene, *bdhA*, encoding 3-hydroxybutyrate dehydrogenase, is important for the long-term survival of *Lp* in water at 37°C (Li et al., 2015).

Due to the success of this approach, we selected another highly up-regulated gene, *lpg1659*, for further characterization. *lpg1659* was significantly up-regulated in *Lp* exposed to water for 2 and 6 h (Li et al., 2015). No previous studies have characterized *lpg1659*, which encodes a hypothetical protein with no putative functions. Nevertheless, we found that this protein is highly conserved in many *Legionella* species, as well as other aquatic bacteria. Therefore, we hypothesized that *lpg1659* is important for *Lp* to survive in freshwater. A deletion mutant of *lpg1659* was used to better understand its role with respect to cell structure, survival in water and growth of *Lp*. We found that Lpg1659 plays a role in the long-term culturability of *Lp* upon water exposure at 37 and 42°C. Evidence presented here suggests that it likely acts as an ion transporter, facilitating the uptake of one or more essential trace metal ions. Based on our results, the *lpg1659* gene was named *lasM* for *Legionella* aquatic survival membrane protein.

## Methods

### Bacterial strains and culture conditions

All *Lp* strains used in this study were constructed from JR32, a streptomycin resistant derivative of *Lp* Philadelphia-1 (Sadosky et al., 1993). The constitutively competent KS79 strain, derived from JR32, was used for the construction of the *lpg1659* mutant strain (de Felipe et al., 2008). Unless specified otherwise, *Lp* was grown on ACES-Buffered Charcoal Yeast Extract agar supplemented with 0.4 mg ml^−1^ L-cysteine, 0.25 mg ml^−1^ ferric pyrophosphate and 0.1% α-ketoglutarate (i.e., BCYEα agar) at 37°C for 3 days (Feeley et al., 1979; Edelstein, 1981). This medium was further supplemented with 25 μg ml^−1^ kanamycin or 5 μg ml^−1^ chloramphenicol, when necessary. *Escherichia coli* strains derived from DH5α were grown on Luria-Bertani agar at 37°C overnight and was supplemented with 25 μg ml^−1^ chloramphenicol, when necessary. Descriptions of the bacterial strains used in this study can be found in Table 1.

**Table 1:** **Bacterial strains used in this study**.

**Name**	**Relevant genotype**	**References**
***LEGIONELLA PNEUMOPHILA***		
JR32	r^−^m^+^, Sm^R^	Sadosky et al., 1993
KS79 (WT)	JR32 Δ*comR*	de Felipe et al., 2008
LELA3118 (*dotA* mutant)	JR32 *dotA*::Tn903dIIlacZ	Sadosky et al., 1993
LM1376 (*rpoS* mutant)	JR32 *rpoS*::Tn903dGent, Gm^R^	Hales and Shuman, 1999
SPF176 (prpoS)	LM1376 pSF49	Trigui et al., 2014
SPF248 (Δ*lasM*)	KS79 Δ*lpg1659*, Kn^R^	This work
SPF294 (Δ*lasM*+plasM)	SPF248 pSF83, Kn^R^Cm^R^	This work
SPF298 (WT+plasMi)	KS79 pSF73, Cm^R^	This work
***ESCHERICHIA COLI***		
DH5α	*supE*44 Δ*lac*U169 (Φ80 *lacZ*ΔM15) *hsdR*17 *recA*1 *endA*1 *gyrA*96 *thi*-1 *relA*1	Invitrogen
pMMB207C	DH5α, Δ*mobA*, Cm^R^	Charpentier et al., 2008
pSF6	DH5α, pGEMT-easy-*rrnb*	Faucher et al., 2011
pSF49	DH5α, pMMB207C-p_tac_-*rpoS*, Cm^R^	Trigui et al., 2014
pSF73	DH5α, pMMB207C-p_tac_-*lpg1659*, Cm^R^	This work
pSF83	DH5α, pXDC39-p*_lpg_1659*-*lpg1659*, Cm^R^	This work
pXDC39	DH5α, pMMB207c, Δ*Ptac*, Δ*lacI*, Cm^R^	Xavier Charpentier

### Construction of mutant, complemented, and over-expression strains

To construct the *lpg1659* deletion mutant strain SPF248 (Δ*lasM*), 1 kb length sequences upstream and downstream of *lpg1659* were first amplified from the wild-type (WT) strain KS79 by PCR using Taq polymerase (Invitrogen), with the primer sets 1659_UpF/1659_UpR and 1659_DownF/1659_DownR, respectively. The kanamycin cassette was amplified from pSF6 with the primer set Kn-F/Kn-R, purified and further amplified with the primer set 1659_KnF/1659_KnR to obtain a 1 kb kanamycin fragment where the 5′ end is complementary to the 3′ end of the upstream fragment, and 3′ end is complementary to the 5′ end of downstream fragment. A 3 kb mutant allele was amplified using the three 1 kb fragments as template, Phusion DNA polymerase (NEB) and the primer set 1659_UpF/1659_DownR. The purified amplicon was then introduced into KS79 through natural transformation (de Felipe et al., 2008). The recombinants were selected for kanamycin resistance, and successful replacement of the target gene by the kanamycin cassette was validated by PCR.

To construct the pSF83 plasmid (plasM) for complementation, the target gene *lpg1659* together with 500 bp region upstream of the translation start site was amplified from KS79 using the primer set Com1659F2_SacI/Com1659R_XbaI. The amplicon and the plasmid pXDC39 were both digested with SacI and XbaI (NEB) and ligated using T4 DNA ligase (NEB). The ligation mixture was transformed into competent *E. coli* DH5α and the transformants were selected for chloramphenicol resistance. Correct insertion of the amplicon in the plasmid extracted from transformants was validated by PCR using the primer set pXDC39-F/Com1659R_XbaI. This pSF83 plasmid was then introduced into the mutant strain Δ*lasM* by electroporation as described previously (Chen et al., 2006), so as to construct the complemented strain SPF294 (Δ*lasM*+plasM). The recombinants were selected for kanamycin and chloramphenicol resistance before validation by PCR.

To construct the pSF73 plasmid (plasMi) for over-expression, the target gene *lpg1659* was first amplified from KS79 using the primer set Com1659F_SacI/Com1659R_XbaI. The amplicon and the plasmid pMMB207c were both digested with SacI and XbaI (NEB) and ligated with T4 DNA ligase (NEB). The ligation mixture was transformed into competent *E. coli* DH5α and the transformants were selected for chloramphenicol resistance. The presence of the insert (*lpg1659*) in the plasmid extracted from transformants was validated by PCR using the primer set PromF/Com1659R_XbaI. This pSF73 plasmid was then introduced into KS79 by electroporation in order to construct the over-expression strain SPF298 (WT+plasMi). The recombinants were selected for kanamycin and chloramphenicol resistance before validation by PCR. The primer sequences are listed in Table 2.

**Table 2:** **Primer sequences used in this study**.

**Name**	Sequence (5′–3′)^*^
1659_UpF	CAATCAGAACAAGGTGTGTATGG
1659_UpR	CAGTCTAGCTATCGCCATGTACGATGAGTACTGAATTCCTGC
1659_DownF	GATGCTGAAGATCAGTTGGGTCACGTCCTATCACATTCTATTACTC
1659_DownR	AGATCGATGAAGGCTTGTAGC
1659_KnF	GCAGGAATTCAGTACTCATCGTACATGGCGATAGCTAGACTG
1659_KnR	GAGTAATAGAATGTGATAGGACGTGACCCAACTGATCTTCAGCATC
1659_QF	CGGTCACTCTTTGGTATATGTC
1659_QR	CTGATTGACTGGATCGAACATC
16s_QF	AGAGATGCATTAGTGCCTTCGGGA
16s_QR	ACTAAGGATAAGGGTTGCGCTCGT
Com1659F_SacI	CCGGAGCTCGCAGGAATTCAGTACTCATCG
Com1659F2_SacI	CCGGAGCTCCACCTTTCAGATTGTTAGTCGC
Com1659R_XbaI	CGCTCTAGAGAGTAATAGAATGTGATAGGACG
Kn-F	TACATGGCGATAGCTAGACTG
Kn-R	ACCCAACTGATCTTCAGCATC
pXDC39-F	GCTTCCACAGCAATGGCATCC
PromF	CGTATAATGTGTGGAATTGTGAG

* The underlined bases indicate restriction sites.

### RT-qPCR

JR32, the *rpoS* mutant and the complemented strain SPF176 were first suspended in ACES-buffered Yeast Extract (AYE) broth at an initial OD_600_ of 0.1. Fifty milliliter cultures of JR32, *rpoS* mutant, SPF176 (prpoS OFF) and SPF176 induced with 0.5 mM IPTG (prpoS ON) were grown in 250 ml Erlenmeyer flasks at 37°C shaking (250 rpm) to exponential phase (OD_600_ of 1.0). Each culture was then centrifuged and washed with Fraquil three times before suspending in Fraquil to an OD_600_ of 1.0. Fraquil is an artificial freshwater medium that does not support growth but allows long-term survival of *Lp* (Li et al., 2015; Mendis et al., 2015). The composition of Fraquil is 0.25 μM CaCl_2_, 0.15 μM MgSO_4_, 0.15 μM NaHCO_3_, 10 nM K_2_HPO_4_, 0.1 μM NaNO_3_, 10 nM FeCl_3_, 1 nM CuSO_4_, 0.22 nM (NH_4_)_6_Mo_7_O_24_, 2.5 nM CoCl_2_, 23 nM MnCl_2_, and 4 nM ZnSO_4_ in ultra-pure Milli-Q water (Morel et al., 1975). Thirty milliliter of each suspension was transferred to 125 ml Erlenmeyer flask and incubated at 37°C shaking for 6 h. Samples were then collected and RNA was extracted as described previously (Li et al., 2015). One microgram of purified RNA was used for reverse transcription reactions along with a negative control without reverse transcriptase. For qPCR reactions, the 16S rRNA gene-specific primer set 16s_QF/16s_QF and the *lpg1659* gene-specific primer set 1659_QF/1659_QR were designed with the IDT primer design software (https://www.idtdna.com/Primerquest/), and their amplification efficiency were proven to be >85% (data not shown). qPCR was performed on an iQ™5 Multicolor Real-Time PCR Detection System (Bio-Rad) using iTaq universal SYBR green supermix (Bio-Rad) according to the manufacturer's protocol. The 16SrRNA gene was used as the reference gene to normalize the data. Fold change was calculated as described previously (Livak and Schmittgen, 2001) and are presented as log_2_ ratios.

### Bioinformatics analysis

The hypothetical protein (accession number: YP_095686.1) encoded by *lasM* was compared to proteins encoded by other bacteria using Standard Protein BLAST (http://blast.ncbi.nlm.nih.gov/Blast.cgi?PAGE=Proteins). The NCBI CD-search was used to identify any conserved domains present in the LasM protein. Three servers from the CBS Prediction Servers (http://www.cbs.dtu.dk/services/) were then used to predict the putative function(s) of LasM. First, TMHMM Server v.2.0 was used to predict transmembrane helices (Sonnhammer et al., 1998; Krogh et al., 2001). Second, SignalP 4.1 Server was used to predict the presence and location of signal peptides (Petersen et al., 2011). Lastly, ProtFun 2.2 Server was used to predict cellular role, enzyme class as well as the gene ontology category based on the amino acid sequence (Jensen et al., 2002, 2003).

### Microscopic examination of cell morphology

The WT strain KS79 and the mutant strain Δ*lasM* were suspended in Fraquil at an OD_600_ of 1.0. Immediately after suspension, a wet mount was prepared and viewed at 1000 × magnification using digital microscopy (Nikon Eclipse 80i). Images of 10 random microscopic fields were captured for each strain using the NIS Element software (Nikon Instruments, Inc.). The length of 10 cells in each image was estimated using the ImageJ software in order to determine the average cell length.

### Extracellular growth assay

The KS79 and Δ*lasM* strains were suspended in AYE broth at an OD_600_ of 0.1. Twenty-five ml of each culture was transferred into three 125 ml Erlenmeyer flasks and grown at 37°C shaking. The OD_600_ of each culture was measured by a spectrophotometer once every 4 h for a period of 32 h.

### Cell lines and infection assays

The amoeba *Acanthamoeba castellanii* was grown to confluence in 20 ml of PYG broth (Moffat and Tompkins, 1992) in a 75 cm^2^ tissue culture flask (Sarstedt) at 30°C. Before infection with *Lp*, the old medium with non-adherent amoebae was replaced with 10 ml of fresh PYG broth. The flask was then shaken sharply to release the adherent amoebae into the medium. This suspension was enumerated and diluted to 5 × 10^5^ cells per ml. One ml was placed into each well of a 24-well plate (Sarstedt). The amoebae were allowed to adhere for 2 h before the medium was replaced with Ac buffer, which does not support the growth of *Lp* (Moffat and Tompkins, 1992). The plate was incubated at 30°C for another 2 h before infection.

The human monocyte-like cell line THP-1 was grown in 30 ml of RPMI 1640 (Life Technologies) supplemented with 10% fetal bovine serum and 2 mM glutamine at 37°C under 5% CO_2_ (Kim et al., 2009). Three days prior to infection, 1 ml of THP-1 culture (5 × 10^5^ cells per ml) was placed into each well of a 24-well plate (Sarstedt) and treated with 1 × 10^−7^ M phorbol 12-myristate 13-acetate (PMA) (Fisher Scientific) to induce maturation toward adherent macrophage-like cells. Subsequently, the medium was replaced by fresh RPMI without PMA 2 h before infection with *Lp*.

For the infection assays, the KS79, Δ*lasM*, and *dotA* mutant were suspended in AYE broth at an OD_600_ of 0.1 and then, diluted 10-fold to approximately 2.5 × 10^6^ cells per ml. The *dotA* mutant is defective for intracellular growth (Roy and Isberg, 1997) and was used as a negative control. Two microliter of each bacterial suspension was added to three replicate wells of *A. castellanii* and THP-1 cells, resulting in an MOI of 0.1. The *A. castellanii* infection plate was incubated at 30°C and the intracellular growth of each strain was determined by CFU counts on BCYEα agar at 24 h intervals for 7 days; whereas the THP-1 infection plate was incubated at 37°C under 5% CO_2_, and the intracellular growth was monitored at 24 h intervals for 5 days.

### Survival assays in water

Strains grown on BCYEα agar were washed three times with Fraquil and suspended in fresh Fraquil at an OD_600_ of 0.1. One milliliter of bacterial suspension was added to 4 ml of fresh Fraquil in a 25 cm^2^ plastic flask (Sarstedt). For each strain, three replicate flasks were incubated at 25, 37, and 42°C, and CFU counts were measured once per 3 weeks, once per 2 weeks and once per week, respectively. In addition, membrane integrity of the samples incubated at 42°C for 7 weeks was determined by Live/Dead staining and flow cytometry, using freshly grown KS79 as the live control and KS79 boiled in a water bath for 10 min as the dead control, as described previously (Li et al., 2015).

For the survival assay using Fraquil containing10 times trace metals, the final salt and trace metal concentration is 0.25 μM CaCl_2_, 0.15 μM MgSO_4_, 0.15 μM NaHCO_3_, 10 nM K_2_HPO_4_, 0.1 μM NaNO_3_, 0.1 μM FeCl_3_, 10 nM CuSO_4_, 2.2 nM (NH_4_)_6_Mo_7_O_24_, 25 nM CoCl_2_, 0.23 μM MnCl_2_, and 40 nM ZnSO_4_.

### Heat tolerance assays

To test tolerance to heat shock, the KS79 and Δ*lasM* strains were suspended in Fraquil at an OD_600_ of 0.1. One ml of each strain was aliquoted into 13 ml tubes (Sarstedt) in triplicate. Tubes were acclimated to 25°C for 24 h and then, transferred into a water bath set to 55°C. Samples were taken before, after 0.5 h and after 1 h of heat shock to determine the changes in CFU counts.

To test growth at an elevated temperature, the KS79 and Δ*lasM* strains were suspended in AYE broth at an OD_600_ of 0.1. Twenty-five milliliter of each culture was transferred into three 125 ml Erlenmeyer flasks and grown at 42°C shaking. The CFU of each strain was monitored daily.

## Results

### *lasM* is positively regulated by RpoS in water

**Figure 1:**
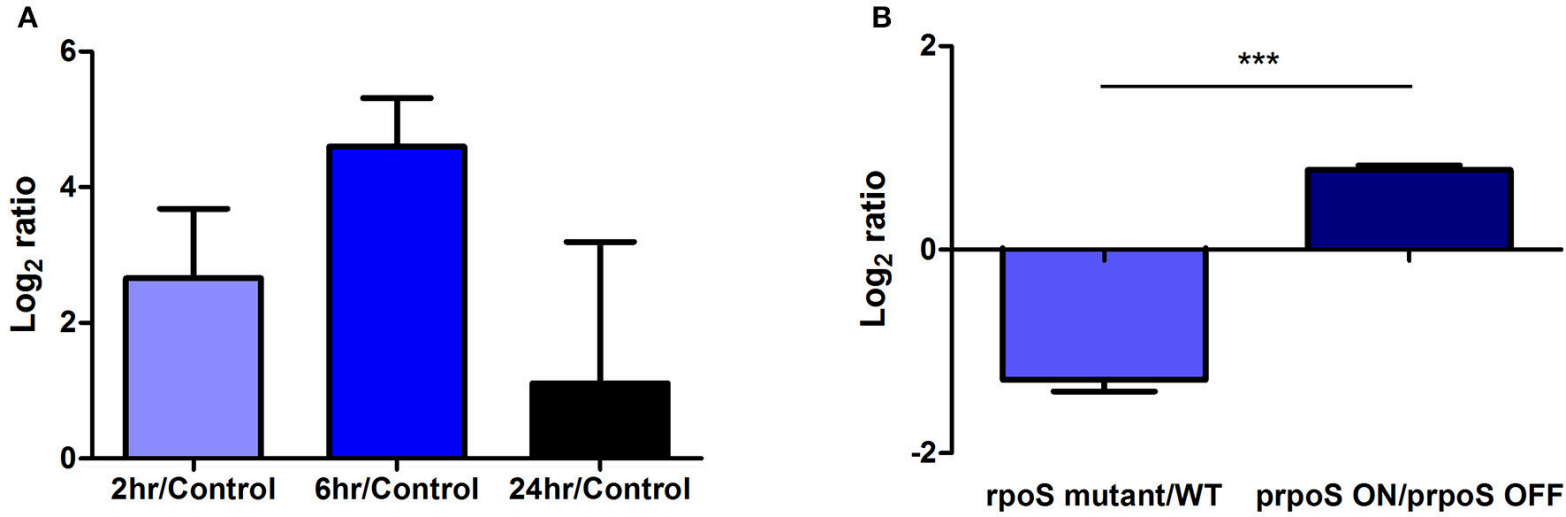
**LasM is expressed in water and is regulated by RpoS. (A)** The expression of *lasM* based on a transcriptomic analysis of the WT JR32 strain exposed to water for 2, 6, and 24 h compared to the control grown to exponential phase in rich medium (adapted from Li et al., 2015 published under Creative Commons Attribution 4.0 International License; https://creativecommons.org/licenses/by/4.0/). **(B)** The RpoS-dependant regulation of *lasM* in *Lp* exposed to water for 6 h was assessed by RT-qPCR. The expression of *lasM* in the *rpoS* mutant was compared to that in the WT strain. The expression of *lasM* in the complemented strain where *rpoS* is induced with 0.5 mM IPTG (prpoS ON) was compared to that of the non-induced complement (prpoS OFF). One-tailed unpaired Student's *t*-test was used to assess significant differences between the two ratios (^***^p < 0.0005). Data shown are the mean and SD of 3 biological replicates.

In a previous study, we found that *lasM* was significantly up-regulated by more than 6-fold after 2 h (Log_2_ ratio = 2.66) and 24-fold after 6 h (Log_2_ ratio = 4.60) of exposure to water when compared to a control grown in rich medium (Figure 1A; Li et al., 2015). RpoS is an alternative sigma factor and a stress response regulator important for the survival of *Lp* in water (Trigui et al., 2014). In order to test whether the expression of *lasM* is controlled by RpoS, RT-qPCR was performed using RNA from the WT, *rpoS* mutant and complemented strains that were exposed to water. The fold change of the *lasM* transcript upon water exposure was lower in the *rpoS* mutant compared to the WT, reflected in the negative Log_2_ ratio of −1.3 (Figure 1B). In contrast, induction of RpoS expression in the mutant (prpoS ON) resulted in a positive Log_2_ ratio of 0.8, indicative of an increase in *lasM* expression, when compared to its non-induced counterpart (prpoS OFF). Taken together, these results suggest that the expression of *lasM* is positively regulated by RpoS in water.

### LasM is a conserved protein found in aquatic bacteria

According to the NCBI Protein database, the hypothetical protein encoded by *lasM* is composed of 347 amino acids. Standard Protein BLAST revealed that LasM found in the Philadelphia-1 strain of *Lp* is highly conserved in other strains, such as Paris, Corby, Alcoy and Lens (≥98% identity). Other *Legionella* species that contain a LasM homolog include *L. norrlandica* (87% identity), *L. moravica* (77% identity), *L. tucsonensis* (76% identity), and *L. longbeachae* (68% identity). LasM also shares significant homology (61–63% identity) with hypothetical or membrane proteins found in *Methylophaga nitratireducenticrescens, Methylophaga lonarensis, Moritella dasanensis, Endozoicomonas elysicola, Colwellia psychrerythraea, Marinobacter santoriniensis* among others. These bacteria were isolated from various aquatic environments, such as water treatment systems, soda lakes and the Arctic Ocean (Kurahashi and Yokota, 2007; Kim et al., 2008; Handley et al., 2009; Antony et al., 2012; Villeneuve et al., 2013). Therefore, the protein sequence homology suggests that LasM is a conserved protein found not only in *Legionella* species, but also in other aquatic bacteria.

**Figure 2:**
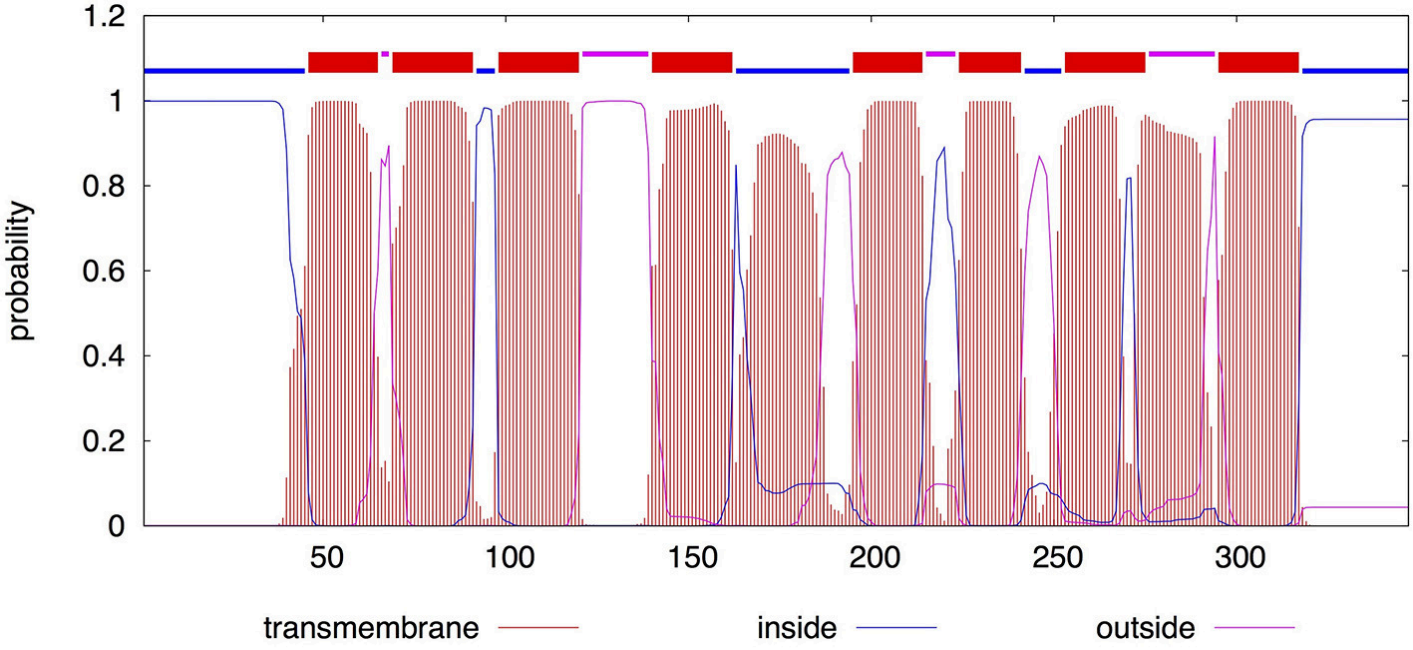
**TMHMM posterior probability for LasM**. The sequence of LasM was analyzed by TMHMM Server v.2.0. The segmented line on top summarizes the most probable location of the sequence. The lower part shows the probability of belonging to different locations. Red indicates transmembrane regions, blue indicates intracellular portions of the protein and pink indicates extracellular portions.

Since LasM does not harbor any conserved domains, *in silico* analysis was performed using the CBS Prediction Servers to predict its putative function(s). Eight transmembrane helices were predicted using the TMHMM server (Figure 2), suggesting that LasM is a transmembrane protein. Moreover, its N-terminal is likely to be located on the cytoplasmic side of the membrane (*p* = 0.99911). No potential signal peptides were identified using the SignalP Server, suggesting that LasM is located in the cytoplasmic membrane. The ProtFun Server predicted LasM to be a non-enzyme (*p* = 0.809) involved in “Transport and Binding” (*p* = 0.740), most likely to be a transporter (*p* = 0.409) among 14 different Gene Ontology categories. Our *in silico* analysis suggests that LasM is a conserved membrane protein, that may be involved in the transport of an unknown substance as part of its aquatic lifestyle.

### Deletion of *lasM* does not affect cellular morphology

**Figure 3:**
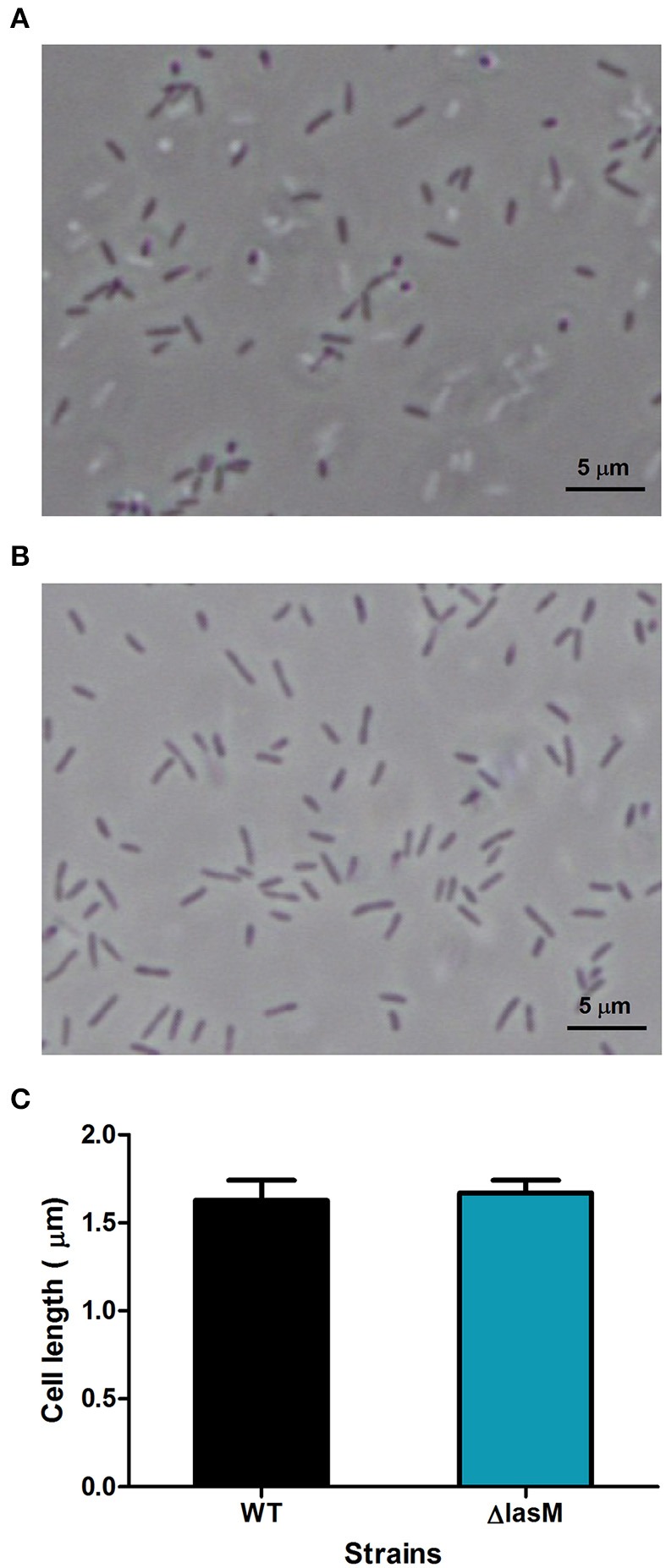
**Deletion of *lasM* does not affect cell morphology or cell size**. A wet mount image of **(A)** the WT strain and **(B)** Δ*lasM* was observed under 1000 × magnification. **(C)** The cell length of the WT strain and Δ*lasM* was estimated using ImageJ. Data shown are the mean and SD of the length of 10 cells per microscopic image in 10 analyzed images (*n* = 100).

Since LasM is predicted to be a membrane protein, we investigated whether the deletion of *lasM* would alter the cellular morphology of *Lp*. Microscopic analysis of wet mounts of 3 days old culture suspended in Fraquil shows that both the WT and the *lasM* deletion mutant are rod shaped cells of comparable size (Figures 3A,B). Statistical analysis confirms that there were no significant differences (*p* = 0.1659) between the cell lengths of the two strains (Figure 3C). These results indicate that the absence of LasM in *Lp* does not affect cell shape or cell size.

### LasM is dispensable for growth *in vitro* and *in vivo*

**Figure 4:**
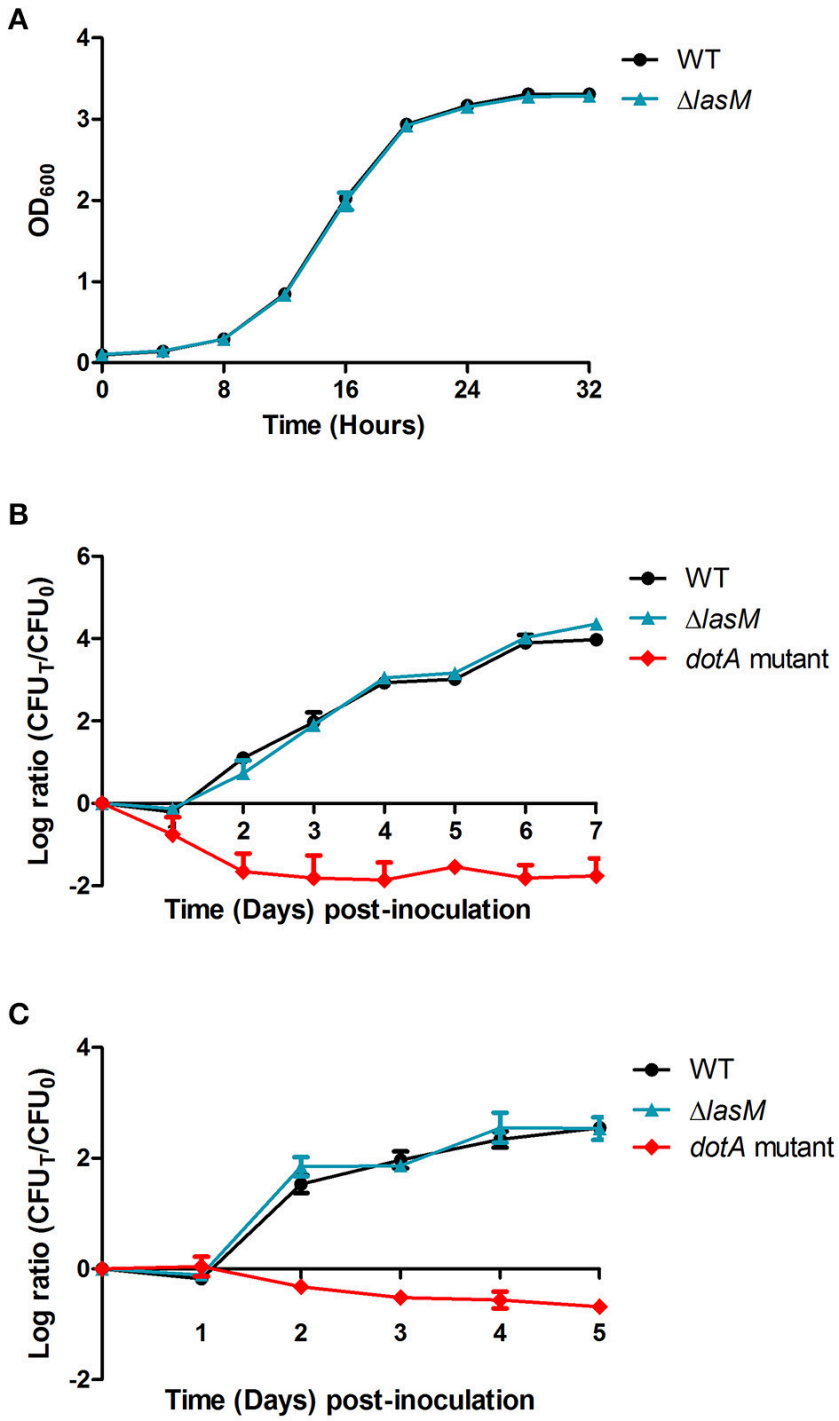
**Deletion of *lasM* does not affect the growth of *Lp in vitro* and *in vivo*. (A)** Optical density at 600 nm of the WT and Δ*lasM* strains grown in rich medium for 32 h. **(B)** The amoeba *A. castellanii* or **(C)** cultured THP-1 macrophages were infected with the WT strain, Δ*lasM* or the *dotA* mutant (negative control) at an MOI of 0.1. Changes in cell titer were monitored using daily CFU counts and are presented as the log ratio of the CFU on each day (CFU_*T*_) over the initial CFU (CFU_0_). Data shown are the mean and SD of 3 biological replicates.

Growth and infection assays were performed to investigate the potential role of LasM in extracellular and intracellular growth. The WT and Δ*lasM* strains produced similar growth curves in rich medium, suggesting that *lasM* is dispensable for the growth of *Lp in vitro* (Figure 4A). The lag phase of both strains lasted 8 h and exponential growth occurred between 8 and 20 h, followed by a late post-exponential/stationary phase.

CFU of the WT and Δ*lasM* strains increased by 4-log after 7 days of growth within the amoeba *A. castellanii* (Figure 4B). In contrast, both the WT and Δ*lasM* only produced a 2.5-log increase in CFU counts after 5 days of growth within human macrophage-like THP-1 cells (Figure 4C). As expected, the *dotA* mutant that served as a negative control demonstrated a reduction in CFU counts in both infection models (Figures 4B,C). No significant differences were observed between the WT and Δ*lasM* in both infection assays, showing that deletion of *lasM* does not affect the ability of *Lp* to infect and multiply intracellularly within *A. castellanii* or within THP-1 cells.

### Deletion and over-expression of *lasM* affects the culturability of *Lp* in water

**Figure 5:**
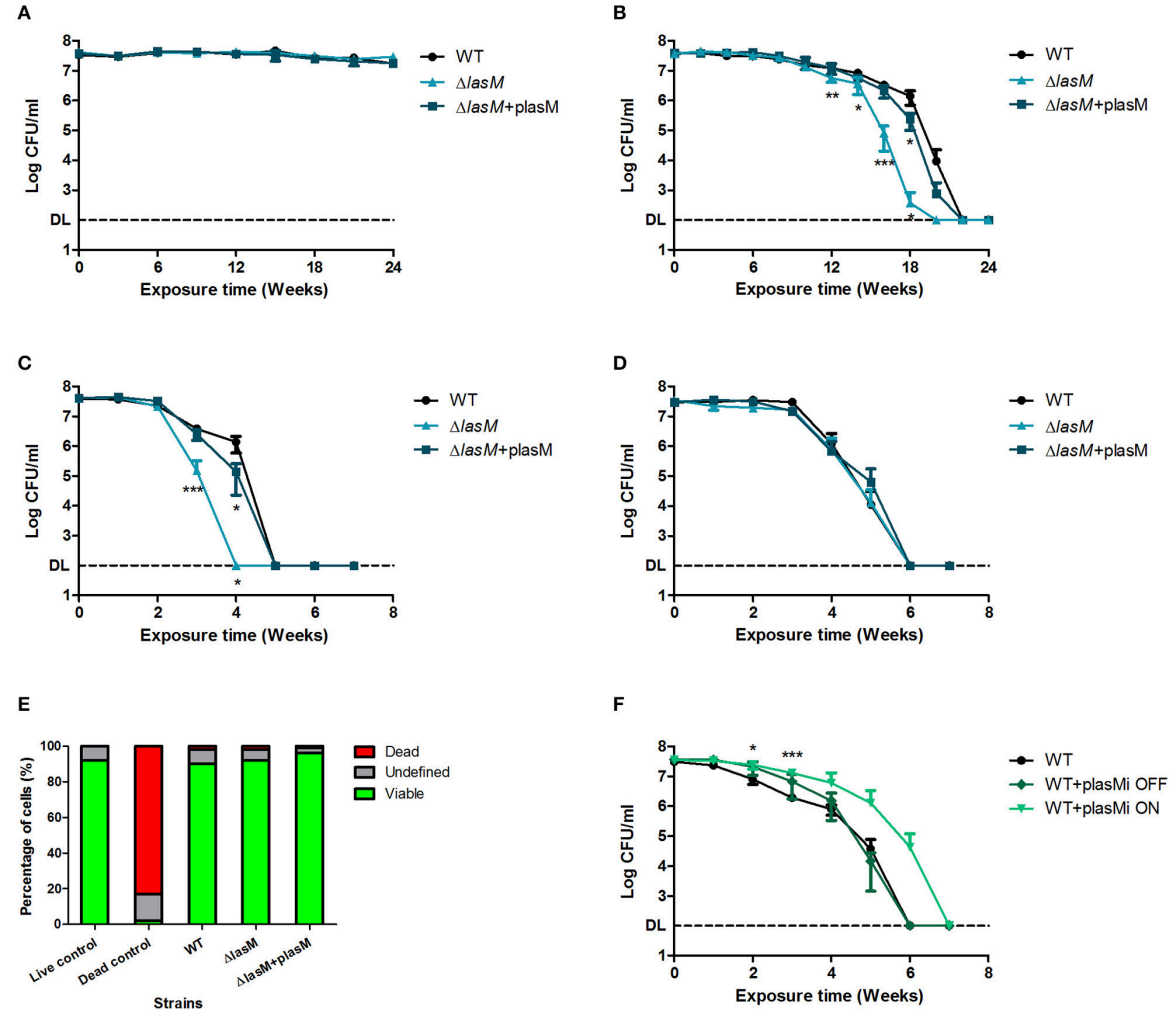
**Deletion and over-expression of *lasM* affects the culturability of *Lp* in water at 37 and 42°C**. The CFU counts of the WT, Δ*lasM* and complemented strain (Δ*lasM*+plasM) in water at **(A)** 25°C, **(B)** 37°C, **(C)** 42°C, and **(D)** in water with 10 times trace metals at 42°C. **(E)** Percentage of cells in different status after exposure to water at 42°C for 7 weeks. Live/Dead staining and flow cytometry were used to analyze 5000 cells in each replicate. Freshly grown *Lp* was used as the live control and heat-killed *Lp* was used as the dead control. **(F)** CFU counts of the WT strain and the over-expression strain (WT+plasMi) in water at 42°C. WT+plasMi OFF indicates that the over-expression of *lasM* was not induced, while WT+plasMi ON indicates that the over-expression of *lasM* was induced with 1 mM IPTG. Data shown are the mean and SD of 3 biological replicates. One-tailed unpaired Student's *t*-test was used to assess significant differences against the WT (^*^p < 0.05; ^**^p < 0.005; ^***^p < 0.0005). DL indicates the detection limit.

Since *lasM* is highly up-regulated in *Lp* exposed to water (Li et al., 2015) and positively regulated by RpoS, we hypothesized that it may be important for the survival of *Lp* in water. We monitored the changes in CFU counts of the WT, Δ*lasM* and the complemented strain (Δ*lasM*+plasM) during long-term exposure to water at three different temperatures. At 25°C, all three strains survived well, and no significant reduction in CFU counts was observed after 24 weeks in water (Figure 5A). In contrast, the CFU of all three strains dropped below detection limit after 22 weeks of water exposure at 37°C (Figure 5B). The Δ*lasM* strain had a faster reduction in CFU count and a significantly lower CFU than the WT starting at week 12. The complemented strain survived as well as the WT, suggesting that *lasM* is indeed important for *Lp* to maintain culturability in water. Similar trend was observed in the strains exposed to 42°C, where an early loss in culturability was only observed in Δ*lasM* but not in the WT or the complemented strain (Figure 5C). At this temperature, the CFU counts of all three strains decreased more rapidly than at 37°C, dropping below the detection limit after only 5 weeks of water exposure.

Since the deletion of *lasM* resulted in an early loss of culturability at both 37 and 42°C, we hypothesized that over-expression of this gene in the WT would promote the culturability of *Lp* in water. Given that the difference in culturability between the WT and Δ*lasM* strains was the greatest at 42°C, the effect of *lasM* over-expression was tested at this temperature. A plasmid containing an inducible P*tac* promoter preceding the *lasM* ORF was constructed and introduced into the WT strain (WT+plasMi). Under non-inducing conditions (WT+plasMi OFF), the decline in CFU counts over time was similar to the WT strain (Figure 5F). Interestingly, over-expression of LasM using IPTG (WT+plasMi ON) increased the culturability of *Lp* by 1 week. This further supports the notion that *lasM* is important for *Lp* to maintain culturability in water.

### Deletion of *lasM* does not affect the culturability of *Lp* in water containing excess trace metals

Given that LasM was predicted to be a membrane protein involved in “transport and binding” and since it was found to be important for maintaining the culturability of *Lp* in water, we hypothesized that this protein could be involved in acquiring essential nutrients that are present in low amounts in water, such as trace metals. Therefore, we tested the culturability of the WT, Δ*lasM* and the complemented strain (Δ*lasM*+plasM) in water containing 10 times of trace metals at 42°C. Surprisingly, the CFU counts declined at the same rate for all three strains throughout the 7 weeks of exposure, and the mutant no longer demonstrated an early loss of culturability as observed previously (Figures 5C,D). Therefore, LasM does not alter the kinetics of culturability over time when excess trace metals are present in water.

### Deletion of *lasM* does not affect membrane integrity of *Lp* exposed to water

After exposure to water at 42°C for 7 weeks, we analyzed the cell status of the WT, Δ*lasM*, and the complemented strain (Δ*lasM*+plasM) using Live/Dead staining and flow cytometry. Live/Dead staining differentiates between dead cells that have a damaged membrane and viable cells with an intact membrane. In this case, over 90% of each strain under investigation stained as viable cells, and less than 2% of each population were stained as dead (Figure 5E). This data shows that the absence of membrane protein LasM does not significantly affect the membrane integrity of *Lp* after exposure to water.

### Deletion of *lasM* does not affect the tolerance of *Lp* to high temperature

**Figure 6:**
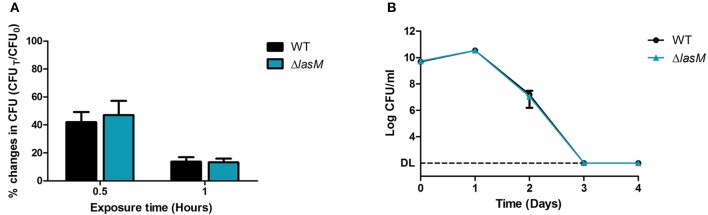
**Deletion of *lasM* does not affect the tolerance of *Lp* to high temperature. (A)** The WT strain and Δ*lasM* were suspended in water and exposed to 55°C for 0.5 and 1 h. The CFU counts after heat shock (CFU_T_) was compared to the CFU counts before heat shock (CFU_0_). **(B)** The WT strain and Δ*lasM* were grown in rich medium at 42°C and their daily CFU counts were monitored. Data shown are the mean and SD of 3 biological replicates. DL indicates the detection limit.

Since early loss of culturability of the *lasM* mutant was only observed at 37 and 42°C but not at 25°C, we investigated whether the mutant was sensitive to elevated temperatures. First, we compared the tolerance of the WT and Δ*lasM* strains in water at 55°C. The CFU counts of both strains decreased to 42–47% of the initial population after 0.5 h and to 13–14% after 1 h of exposure to heat shock (Figure 6A). Increasing exposure time significantly reduced their CFU counts (*p* < 0.0001), but no significant differences were found between the two strains (*p* = 0.5605). Then, we compared the tolerance of both strains in rich medium at 42°C and, again, found no significant differences between their CFU counts over a period of 4 days (Figure 6B). Both strains grew in the first day and then their CFU counts dropped below the detection limit after 3 days. Taken together, these results suggest that deletion of *lasM* does not affect the tolerance of *Lp* to high temperature in water or in rich medium.

## Discussion

Despite the lack of nutrients, *Lp* can survive in freshwater for a prolonged period of time and also remain infectious (James et al., 1999; Li et al., 2015; Mendis et al., 2015). In this study, we characterize *lpg1659* (*lasM*), a gene that encodes a hypothetical protein with no putative functions. *In silico* analysis shows that *lasM* encodes a membrane protein that is likely involved in transport and binding. Differential expression of membrane proteins are well documented in literature. In *E. coli*, some membrane proteins like OmpA and general porins are constitutively expressed at high levels, whereas other membrane proteins are only expressed under specific conditions (Koebnik et al., 2000). Exposure to water at varying temperatures elicited specific changes in the outer membrane protein composition of enterohemorrhagic *E. coli* (Wang and Doyle, 1998). In another waterborne pathogen *Leptospira interrogans*, the inner membrane protein LipL31 was specifically down-regulated during exposure to distilled water, while other membrane proteins were not affected (Trueba et al., 2004). In our case, the expression of the LasM membrane protein was induced significantly after 2 and 6 h of exposure to water, compared to exponential growth in AYE (Li et al., 2015). A previous study showed that *lasM* is also induced in the post-exponential phase of growth (Hovel-Miner et al., 2009). Therefore, it is likely that a signal present in Fraquil and post-exponential phase, such as starvation, induces the expression of *lasM*. Since *Lp* shuts down transcription and translation 24 h after exposure to water (Trigui et al., 2014; Li et al., 2015), genes required for survival in water are likely induced during earlier time points (i.e., the adaptation period), and are subsequently repressed. For example the *bdhA* gene, encoding 3-hydroxybutyrate dehydrogenase, is required for the long-term survival of *Lp* in water, and follows this expression pattern (Li et al., 2015).

Since the alternative sigma factor RpoS is essential for *Lp* to survive in water (Trigui et al., 2014), any genes under the control of RpoS could also be involved in the survival of *Lp* in water. For example, *bdhA* is positively regulated by RpoS in water and it was found to be important for the survival of *Lp* in water at 37°C (Li et al., 2015). According to a previous study, *lasM* is positively regulated by RpoS in rich medium (Hovel-Miner et al., 2009). Here, we report that *lasM* is also under RpoS control when *Lp* is exposed to water, further suggesting an association between LasM and water survival.

Since LasM is predicted to be a membrane protein, we hypothesized that it may play a role in maintaining cell morphology or membrane integrity. However, deletion of *lasM* affected neither the cell shape nor size of freshly grown *Lp* that was suspended in water. Moreover, the membrane integrity of *Lp* exposed to water for 7 weeks was unaffected. We further found that deletion of *lasM* does not affect the ability of *Lp* to infect host cells such as *A. castellanii* and THP-1 cells. Furthermore, it does not affect the growth of *Lp in vitro*. This is consistent with a previous study showing that the insertion of a transposon in the *lasM* gene did not result in growth advantages or disadvantages in rich medium (O'Connor et al., 2011).

Nevertheless, we observed an early loss of culturability in Δ*lasM* exposed to water at 37 and 42°C, suggesting that *lasM* is important for *Lp* to maintain culturability in warm water. These temperatures are commonly found in man-made water systems such as cooling towers that *Lp* is able to colonize (Rogers et al., 1994; Darelid et al., 2002). Live/Dead staining shows that the proportion of dead cell in the Δ*lasM* population is comparable to that of the WT, but the mutant enters a viable but non-culturable (VBNC) state earlier than the WT. VBNC cells are in a quiescent state awaiting revival or transitioning to death (Li et al., 2014). Previous studies show that *Lp* that was induced into the VBNC state under certain conditions, such as starvation and exposure to disinfectants, may be resuscitated back into culturable and infectious cells using different methods (reviewed by Li et al., 2014). For example, *Lp* that entered the VBNC status 125 days after exposure to sterilized tap water at 20°C were resuscitated by the addition of *A. castellanii* (Steinert et al., 1997). However, our attempt to resuscitate the samples using the same method failed and the VBNC cells remained non-infectious (data not shown). Differences in VBNC-inducing conditions (e.g., water, temperature, etc.) and additional factors may contribute to the failure of resuscitation (reviewed by Li et al., 2014). VBNC cells that cannot be resuscitated are considered to be in the process of dying. Therefore, we conclude that Δ*lasM* started dying at an earlier time point than the WT when exposed to water. Given that over-expression of LasM also promotes the culturability of *Lp* in water, we conclude that LasM is important for the long-term survival of *Lp* in water at temperatures above 25°C.

Early loss of culturability was not observed in Δ*lasM* exposed to water at 25°C for 24 weeks. This is mirrored in a previous study where the deletion of *bdhA* results in an early loss of culturability and causes a survival defect in *Lp* exposed to water at 37°C but not at 25°C (Li et al., 2015). In both cases, it is possible that the survival defect becomes apparent at 25°C after a longer incubation period in water. Since CFU counts drop more gradually at 25°C than at 37 and 42°C (Mendis et al., 2015), any defect resulting from the deletion of an important gene may appear at a later time point at 25°C.

Environmental stresses such as heat shock are known to increase membrane fluidity and eventually result in cell damage (Beney and Gervais, 2001; Richter et al., 2010). Therefore, it is possible that LasM is important for *Lp* to maintain culturability at a higher temperature, not necessarily in water. If the absence of the LasM membrane protein reduced the ability of *Lp* to deal with heat-induced membrane damage, the mutant would produce a faster drop in CFU counts than the WT at elevated temperatures. However, we show that deletion of *lasM* does not affect the rate of CFU reduction in *Lp* exposed to water at 55°C or the CFU changes in *Lp* grown in rich medium at 42°C, suggesting that LasM is important for maintaining culturability in water, but that it is not directly involved in the resistance of *Lp* to high temperatures.

It is noteworthy that LasM was predicted to be a transporter, albeit with low probability (*p* = 0.409). Since the metabolic rate of *Lp* increases with increasing temperature (Kusnetsov et al., 1996), more energy and resources would be needed for active metabolism at higher temperatures. Therefore, if the function of LasM is to facilitate nutrient transport, then the loss of LasM could result in a more severe defect in water at 37°C or at 42°C than at 25°C. Indeed, the absence of LasM did not affect the kinetics of culturability in water with 10 times the original amount of trace metals, suggesting that the early loss of culturability previously observed in the mutant can be complemented by excess trace metals in water, namely copper, molybdenum, cobalt, manganese, zinc, sodium and iron. This finding supports our hypothesis that LasM is a transporter of one or more of the essential trace metals present in water. In the absence of this transporter, *Lp* might not be able to acquire sufficient trace metals from water environment to maintain culturability, and possibly long-term survival. Our data show that over-expression of *lasM* in *Lp* seems to allow better acquisition of essential trace metals, helping to maintain culturability for a longer period of time. Extra trace metals in water might also increase the amount that diffused into cells, and thus, allow the mutant lacking LasM to maintain culturability as well as the WT. It is not yet clear which trace metal is transported by LasM. Based on our experiments, LasM could transport one or a combination of copper, molybdenum, cobalt, manganese, zinc and iron.

In conclusion, this study reveals that the LasM protein is important for *Lp* to acquire essential trace metals in order to maintain culturability in water, which is consistent with its most probable predicted function. Our results do not ruled out the possibility that LasM could increase the fitness of *L. pneumophila* in other settings where the concentration of metals is low or where there is fierce competition for them, such as multi-species biofilms existing in water systems. The *lasM* gene is highly up-regulated in water and positively regulated by RpoS. It encodes a novel membrane protein, which is highly conserved in many *Legionella* species and other aquatic bacteria. We postulate that LasM is an important protein for other aquatic bacteria to maintain culturability and survival in water and in conditions presenting low concentration of metals. Absence of this protein does not affect cell morphology, membrane integrity, tolerance to high temperature or the growth of *Lp*, both *in vitro* and *in vivo*. Further investigation would be required to better understanding the exact trace metal(s) being transported by LasM and the underlying mechanism.

## Author contributions

LL and SF conceived and designed the experiments. LL conducted the experiments and wrote the manuscript. SF contributed in writing and review of the manuscript.

## Funding

This work was supported by the NSERC Discovery grant 418289-2012 and John R. Evans Leaders Fund—Funding for research infrastructure from the Canadian Foundation for Innovation to SF.

### Conflict of interest statement

The authors declare that the research was conducted in the absence of any commercial or financial relationships that could be construed as a potential conflict of interest.

## Abbreviations

*Lp*: *Legionella pneumophila*
VBNC: viable but non-culturable
WT: wild-type.
